# Dynamic swatch testing of liquid aerosols in a laboratory-sized recirculating wind tunnel

**DOI:** 10.1038/s41598-024-67643-0

**Published:** 2024-07-17

**Authors:** Seung Jung Yu, Goonhyeok Kim, Hyunsook Jung, Heesoo Jung, Jaewuk Jung, Daegyoum Kim

**Affiliations:** 1 Chem-Bio Technology Center, Agency for Defense Development, Yuseong-Gu, Daejeon, 34063 Republic of Korea; 2 Weapon Systems Engineering, University of Science and Technology, Gajeong-Ro, Yuseung-Gu, Deajeon, 34113 Republic of Korea; 3 Department of Mechanical Engineering, KAIST, Daejeon, 34131 Republic of Korea

**Keywords:** Dynamic, Swatch, Liquid aerosol, Wind tunnel, Penetration, Environmental sciences, Engineering, Materials science

## Abstract

Chemical warfare agents (CWAs) pose a threat as gaseous substances and as liquid aerosols, necessitating chemical warfare-protective clothing for soldiers. The paramount consideration lies in the effectiveness of the clothing as a barrier against the pertinent CWAs. This paper presents a dynamic swatch test method aimed at evaluating the performance of such clothing against liquid-phase aerosol penetration. Central to the methodology is a specialized test cell designed to rotate to the left and right, integrated within a laboratory wind tunnel, replicating mission-relevant conditions with varying wind speeds. Utilizing di(2-ethylhexyl) sebacate particles as liquid aerosols, tests were conducted at wind speeds of 1.0, 3.0, and 5.0 m/s. Penetration assessment relied on analyzing particle counts downstream and upstream of the fabric, with preliminary studies showing that higher wind speeds and fabric air permeabilities increase penetration at an equivalent face velocity of 5.0 cm/s. Interestingly, penetration decreased when fabric samples were subjected to rotation. The system and methodology devised demonstrated consistent and repeatable results, offering valuable insights into optimizing the effectiveness of chemical warfare-protective clothing. This research contributes to advancing methodologies for testing protective clothing, crucial for ensuring the safety of military personnel in hazardous environments.

## Introduction

In assuming the risk of exposure to chemical warfare agents (CWAs) on the battlefield, soldiers are required to wear chemical warfare-protective clothing. CWAs are commonly produced in gaseous phases and as liquid aerosols. Harmful biological warfare agents such as anthrax also exist in aerosol form. Therefore, understanding the performance of chemical warfare-protective clothing against harmful CWA gases and dermal exposure to hazardous CWAs in the liquid aerosol phase is crucial. Generally, the fabric used for a soldier’s protective clothing consists of an outer tightly woven layer and an activated carbon layer^1^. Harmful gases are captured by the activated carbon adsorbent, while the fibers, particularly in the outer woven layer of the fabric, provide most of the airflow resistance and particulate filtration. However, aerosols can be carried by air flows and the outer woven layer does not capture all the particles transported by the air flowing through it, posing health hazards through dermal exposure^2^. The toxicity of these aerosols is usually a function of their hazardous constituents or evaporation and recondensation^3^.

Dedicated standards exist to certify the protective value of such clothing, but due to the differences in assessment and specific requirements for target products, the relevant test methods for such protective clothing performance vary greatly^4,5^. In particular, the test standard for civilian chemical protective clothing providing protection against airborne solid chemicals (Type 5) offers a relatively realistic test protocol. This method involves generating a standard aerosol in a test chamber, where a test subject wearing the chemical protective suit carries out a sequence of predetermined test exercises, and measures the inward leakage into the protective clothing^6^. The aerosol man-in-simulant test (MIST) has been the primary method for testing the penetration of aerosol simulants through chemical warfare-protective clothing^7^. In the aerosol MIST, a human subject wears a complete chemical warfare-protective clothing ensemble in a test chamber while being exposed to a fluorescent-tagged silica aerosol simulant. The participant performs a motion routine simulating a real-life scenario, and after the trial, the amount of fluorescent tracer present on the participant’s skin at various locations is analyzed. These measurements are then used to determine a relative protection rating for the ensemble. However, both standards require human participation as subjects, exposing them to chemical simulants such as sodium chloride or fluorescent silica particles (dry, solid particles) during the test, thus necessitating careful risk assessment and approval in accordance with FDA regulations^7^.

As the interaction of fabrics with particles resembles that of filter media, studies have investigated this phenomenon by performing classical filtration experiments with fabrics. In these experiments, aerosol particles are pulled through a clothing sample while monitoring the particle concentration upstream and downstream^8,9^. Similarly, studies on particle penetration through chemical warfare-protective clothing materials have often been carried out following standardized filter test methods^10^. In these filtration-based test methods, a piece of fabric or swatch is placed in a closed environment and, air is forced through the material, and the upstream and downstream aerosol concentrations are evaluated to assess the degree of particle penetration. However, these methods may not reflect mission-relevant conditions under which the protective clothing operates in the field, where aerosol penetration is primarily caused by wind and body movement^11,12^. Aerosol particles carried with air currents can be transported across the porous structures of air-permeable fabrics, and the user, acting as a blunt body behind protective clothing, creates shifts in airflow streamlines, altering the penetration of particles through the ensemble^13^.

Recognizing the limitations of classical filtration approaches in simulating the field use of protective clothing, some authors have developed experimental setups to study particle penetration of particles^2,11,14–16^. For instance, Bergman et al. developed a test technique mirroring the process of wind-driven aerosol transport through outdoor clothing^14^. The wind was generated to emulate the outdoor environment as closely as possible. A cylindrical test cell representing a segment of a person’s arm, leg, or torso was used to attach a test cloth and air was pulled through the fabric. Gao et al. created a cone-shaped penetration cell, which was placed in a wind tunnel where the face velocity was generated through the clothing by a wind-driven method^11^. The authors found that the penetration values recorded using the wind-driven approach were lower than those obtained using the filtration method for most studied textiles. The relative penetration performance of the fabrics varied considerably due to wide variations in pore structure. While this setup could mimic real exposure situations, adjustments were made to the wind speed to reflect the classical filtration approach at low fabric velocities. More recently, Hill et al. studied the penetration of liquid aerosol particles into a fabric stretched over a vertical tube with proportions comparable to those of a human arm, considering the annular space that resembled the air space between loose-fitting clothing and skin^2,15^.

The effect of bellows should also be considered when understanding the effectiveness of protective clothing in preventing the ingress of aerosols^17^. When moving, airflow occurs in and out of gaps or holes in materials or ensembles, such as in the closures, seams, or areas at the ankles, wrists, and neck. When worn, clothing is subjected to pressure differentials across the garment from wind, breathing, or the movement of the person wearing it, forcing CWAs vapors or aerosols through the clothing fabric and closures. The bellowing action allows for cooling air circulation, which might be welcome in many circumstances but not when working with hazardous substances such as CWAs, as they can be drawn into any gaps or holes and penetrate onto the body due to the bellows effect.

This study creates a dynamic swatch test method mainly aiming at studying the influence of wind orientation on aerosol penetration through any kind of fabrics. The sample holder, swatch test cell was designed to have a geometrical shape^12^ and to rotate left and right, which could simulate conditions facing the wind or sideways to the wind and thus study the effect of wind orientation. Moreover, the test cell was set up in a laboratory-sized recirculating wind tunnel, emulating real battle conditions as closely as possible. As a proof of concept, the performance of fabric pieces, currently in use by the military as chemical warfare-protective clothing, was tested in the swatch test cell. The penetration characteristics of di(2-ethylhexyl) sebacate (DEHS) aerosols were investigated using different wind speeds, cell rotations, and swatches. These performances were analyzed based on fabric characterization and the filtration theory^18^.

## Results and discussion

### Dynamic swatch test in a laboratory-sized recirculating wind tunnel

As a preliminary validation of the dynamic swatch test in a laboratory-sized recirculating wind tunnel, initial penetration experiments were conducted in the dynamic swatch cell under near-isokinetic conditions without swatch fabric to determine whether consistent changes occurred in the upstream and downstream measurement systems^19,20^.

**Figure 1:**
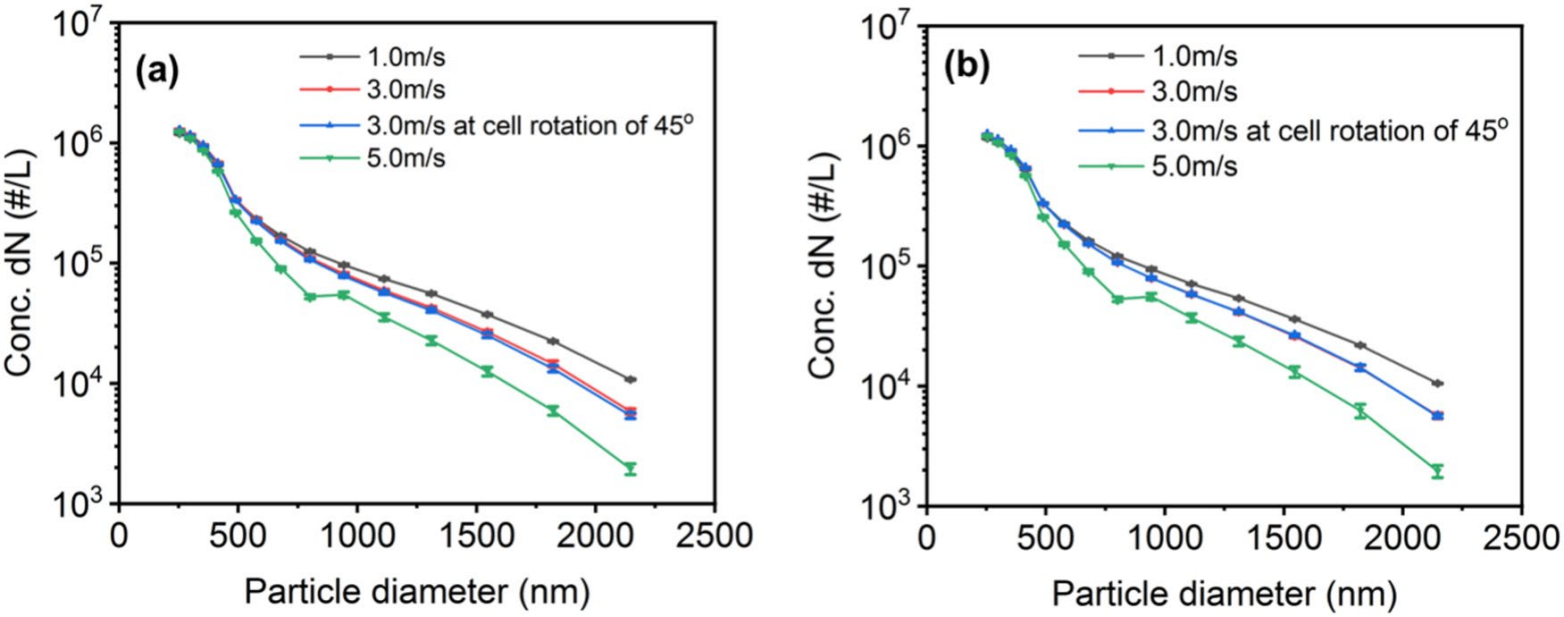
Typical test repeatability of the (**a**) upstream and (**b**) downstream samples. The raw data are provided in the Supporting Information (S1).

Figure 1 shows typical upstream and downstream concentrations expressed in units of dN, where N is the number of particles using DEHS as the test aerosol. Wind tunnel speeds of 1.0, 3.0, and 5.0 m/s were used. An additional experiment was performed at 3.0 m/s at a cell rotation of 45°. It was confirmed that when there was no swatch fabric, the penetration was close to 100% on average for 14 particle sizes ranging from 250 to 2100 nm for all conditions (the average number of particles in the upstream and downstream is given in the Supporting Information, S1). The data were repeatable, and there were no discrepancies in the upstream and downstream data due to the particle distribution.

Notably, exact isokinetic conditions could not be achieved for all experiments. The velocities of the sampling probes upstream and downstream were calculated to be approximately 3.0 m/s (the flow rates of the sampling probes were set to 1.2 LPM by the GRIMM analyzer and the inner diameter of the probes was 3 mm). It is also noted that the sampling probe in the upstream was pumped by the GRIMM analyzer. Therefore, in these experiments, isokinetic conditions may change at the upstream probe with the changing wind speeds. In fact, the sampling rate upstream may be slower at a wind speed of 5.0 m/s or faster at a wind speed of 1.0 m/s, while the downstream probe can capture almost all the particles that pass through.

It should be noted that multiple measurements of background particles were taken throughout the testing procedure, and the data were consistently below ten counts for particle sizes from GRIMM, and well below that in most cases. There was no long-term change in these baseline values. Since this is a novel approach for evaluating swatch components, it was necessary to evaluate the robustness of the test setup and methodology, which conditions left the system ineffective, and the reproducibility of the results.

### Particle-size-dependent fabric penetration

**Figure 2:**
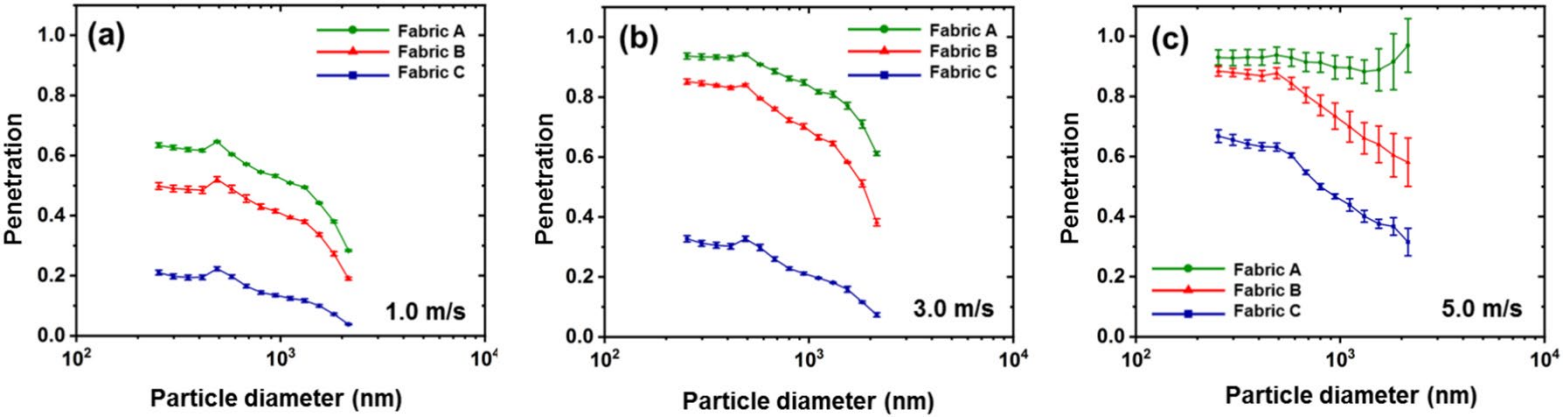
Di(2-ethylhexyl) sebacate (DEHS) particle penetration across the fabrics was measured at different wind speeds with a constant face velocity of 5 cm/s. (**a**) 1.0 m/s, (**b**) 3.0 m/s, and (**c**) 5.0 m/s. The statistical analysis results are provided in the Supporting Information (S9).

Free stream wind speeds, fabrics, and their rotation were used to characterize fabric penetration as a function of particle size. Figure 2 shows the results of the dynamic measurement of the penetration of fabric swatches at three different wind speeds in the free stream.

The data showed that the free stream wind speed significantly affected particle penetration. The particle penetration for all the tested fabrics increased when the wind speed increased from 1.0 to 5.0 m/s at a constant tissue surface velocity of 5.0 cm/s. Among the three fabrics tested, the penetration of Fabric B was the most responsive to the wind speed in all particle size ranges. When the wind speed increased from 1.0 to 3.0 m/s, the penetration levels of the ~ 500 nm particles increased from 50 to 85%. According to single-fiber filtration theory, the filtration behavior of a fabric is characterized by three main parameters, namely, the fiber diameter, fabric thickness, and fabric porosity, which represent the behavior of an ideal fabric^21^. It also strongly depends on the particle characteristics, flow regime, and transport mechanism^22^. Fabric B had the relatively larger fiber diameter, which may affect its filtration behavior with varying free stream wind speeds to a lesser extent ^18,23^. Fiber size is related to aerosol transport and deposition^24^. In fact, larger fiber diameter provides smaller cumulative surface area for aerosol deposition. Another factor related to fiber diameter is interception. The single-fiber penetration due to interception is approximately proportional to the ratio of the particle diameter to the fiber diameter^24^. Considering the effect of adjacent fibers, complex interactions between these factors may be attributed to the penetration characteristics of Fabric B.

The data also showed that Fabric A and B had moderate to high penetration, while Fabric C had substantially less penetration than the other fabrics. Fabric C penetration ranged from less than 10% for the largest particles to no more than 40% for the smallest particles. Notably, among these three fabrics, significantly different properties were observed in terms of thickness and air permeability (Table 1 and Table S2). Indeed, Fabric C had the greatest thickness, and Fabrics A and C differed in terms of air permeability by approximately an order of magnitude (3.78 and 0.54 cm^3^/cm^2^/s at 25 Pa, respectively). In principle, aerosol penetration decreases multiplicatively with increasing filter thickness and decreasing air permeability because there are more materials providing sites for aerosol deposition, although the shape of the penetration curve should remain the same^24^. The limited particle penetration of Fabric C could be mainly attributed to its large thickness and low air permeability, which compensate for its relatively large pore size.

**Table 1:** Summaries of fabric characteristics.

	Fiber diameter (μm)	Thickness (μm)	Porosity	Air permeability (cm^3^/cm^2^/s)	Pore volume × 10^−3^ (m^3^/kg)	Pore size (radius, μm)
Fabric A	13.9 (± 0.54)	365 (± 7.68)	0.5841 (± 0.004)	3.78 (± 0.17)	0.94 (± 0.02)	11.65 (± 0.09)
Fabric B	15.4 (± 0.62)	385 (± 8.34)	0.5845 (± 0.003)	1.89 (± 0.02)	0.98 ((± 0.01)	12.92 (± 0.06)
Fabric C	13.6 (± 0.38)	410 (± 9.21)	0.5841 (± 0.007)	0.54 (± 0.01)	1.04 (± 0.03)	11.40 (± 0.17)

As is typical in fibrous filters, the penetration decreased with relatively large particles (> 500 nm), presumably due to inertial effects^25^, and for smaller particles (< 300 nm) the penetration increased slightly^26^. Notably, the highest penetration was 500 nm for all the tested fabrics. Based on the filtration theory, the most penetrating particle size (MPPS) is affected by filter or fabric properties (fiber diameter, thickness, air permeability, porosity, and pore size, etc.), wind speed and the face velocity^15,24,27^. Jung et al. shows that the MPPS decreases as face velocity increases in the theoretical studies, which is attributed to the increased impaction mechanism at higher velocity and, thus decreased Brownian motion^28^. Huang et al. reported, in contrary, that the MPPS increases with increasing fiber diameter and face velocity^24^. In fact, the MPPS is a function of particles, flow, and filter parameters that do not alter the filtration with the same magnitude^15^. The filter thickness, density, or fiber charge density would cause the MPPS to decrease or increase.

**Figure 3:**
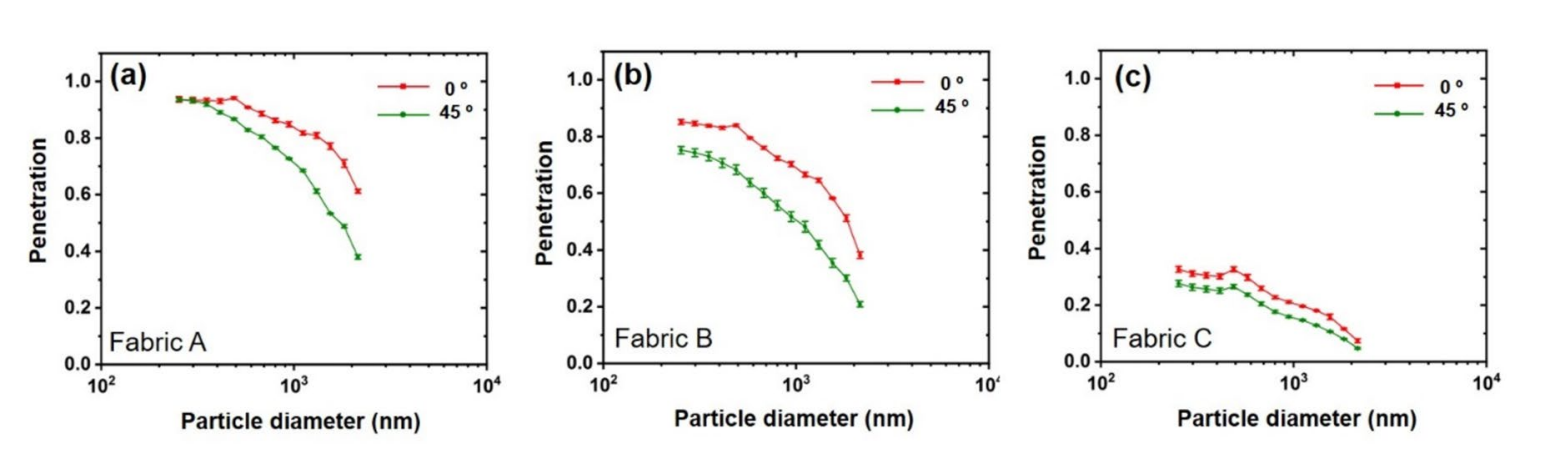
Dynamic swatch test results for two distinct cell rotation angles, 0° and 45° for 3 m/s wind speed and 5.0 cm/s face velocity. (**a**) Fabric A, (**b**) Fabric B, and (**c**) Fabric C. The statistical analysis results are provided in the Supporting Information (S10).

All three fabrics were subjected to dynamic swatch tests at two distinct cell rotation angles, 0° and 45°, at a wind speed of 3.0 m/s. During the testing, the face velocity was set to a value corresponding to 5.0 cm/s. As shown in Fig. 3, a 45° cell rotation resulted in a lower penetration for all three fabric swatches than a 0° cell rotation. Practically, there is no difference downstream for the average total number of particles at a wind speed of 3.0 m/s in both the 0° and 45° cell rotations (Fig. 1b and Fig. S1). Moreover, CFD analysis showed that particles flow perpendicular to the test cell in both 0° and 45° cell rotations in the presence of swatch fabric (S3). These results demonstrate that the sampling probe downstream captures particles equally in both 0° and 45° cell rotations, indicating that the 45° cell rotation does not reduce the sampling probe efficiency downstream.

It should also be noted that Fabrics A and B were far more sensitive to cell rotation than Fabric C. In fact, the effect of cell rotation on the penetration is expected to be due to both air permeability of the test fabric and the size of the air space^29^. When the cell is rotated, the effective surface area of the fabric swatch for the aerosol penetration could be more responsive to the air permeability. Fabrics A and B have relatively high air permeabilities. As a result, the greater the air permeability of the fabric was, the greater the magnitude of penetration and thus the greater the effect of cell rotation.

It should be noted that the chemical warfare-protective clothing was used a single time for each experiment. However, the clothing is meant to be used multiple times after laundry or sterilization, considering the economy or lack of availability. Depending on a variety type of usage condition, the charge generated on aerosols could affect the penetration performance^30^.

## Conclusion

A dynamic swatch test cell was designed to rotate left and right, allowing to test the influence of wind orientation on filtration performances in the cell. The penetration of DEHS liquid aerosols into three different fabrics ranging in size from 250 to 2100 nm was tested at various wind speeds and with cell rotation. The data indicate that increasing the wind speed led to greater particle penetration when the face velocity was constant, demonstrating particle size dependence. Fabric properties and cell rotation were also found to affect the results. The system proved reproducible, with consistent and repeatable results. This novel technique incorporating cell rotation, could enable swatch-level testing to correlate with systematic testing of individual components of protective systems, such as closures, seams, and fasteners, to enhance system performance.

Quantifying or modeling the risk associated with aerosols released outdoors and carried by the wind can inform fabric development programs for individual protective equipment and enable reassessment of aerosol protection requirements. Moreover, applying such a refined test method to CWA protective-fabrics may offer additional trade-offs, such as notably enhanced chemical warfare aerosol protection.

Finally, we noted that the dynamic test cell used in the study was relatively small, and thus, only relatively small fabric swatches can be used. More complex fabric pieces with stiches, taped, or heated-welded seams and gaps cannot be tested. In fact, aerosols tend to take the easiest route of entry. They will more readily and quickly penetrate through holes or gaps rather than permeate through the fabric. For further investigation, we have recently created large-sized dynamic test cells in 2D and 3D (a cylindrical shape). A large donut-shaped wind tunnel, capable of accommodating these large cells has also been constructed. We are currently conducting additional experiments in this larger setup to further investigate the penetration based on cell rotations and fabric characteristics. The results will be presented in future publications.

## Methods

### Swatch test fabrics

Three woven textiles were used for this study: polyester/cotton (68/32) (Fabric A), polyester/cotton (35/65) (Fabric B), and nylon/cotton (50/50) (Fabric C). These fabrics (A, B, and C) are currently being employed as chemical warfare-protective clothing. Therefore, their level of particle penetration is expected to cover a wider range than normally expected for filter materials and allow for a more accurate evaluation of the dynamic swatch test procedure.

**Figure 4:**
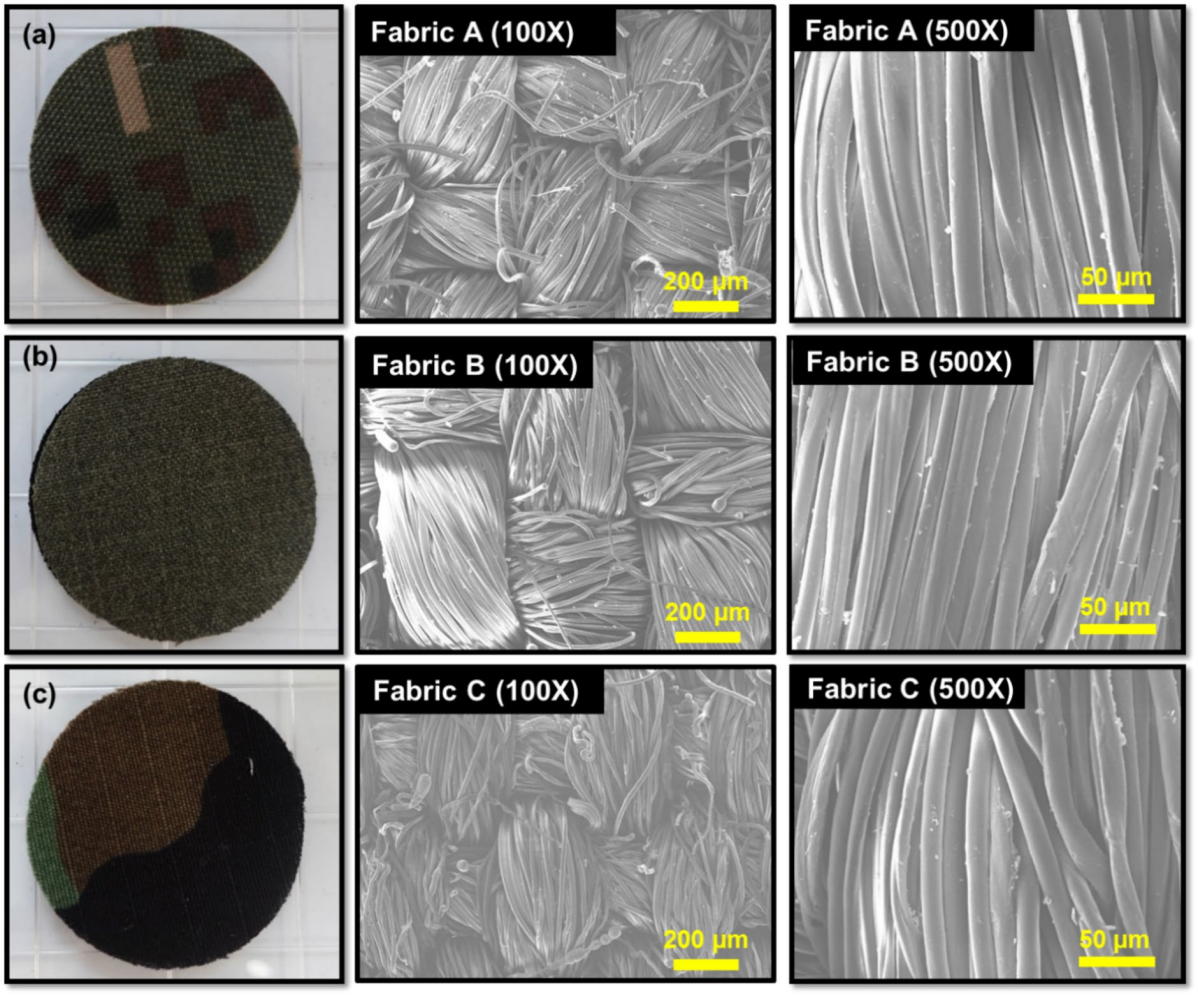
Optical images and scanning electron microscopy (SEM) images at 100× and 500× magnifications of (**a**) Fabric A, (**b**) Fabric B, and (**c**) Fabric C.

To better understand the interaction of particles with fabrics and improve the interpretability of the results, the physical properties of the test fabrics, such as fiber diameter, fabric thickness, air permeability, porosity, pore volume, and pore size, were characterized^11^. Triplicate samples were used for each fabric property measurement. The diameters of the fibers were determined using a scanning electron microscopy (SEM) (JSM-IT500HR; JEOL, Japan) set to 5 kV accelerating voltage and 35 A probe current. At least 50 readings were collected for each fabric sample, and the results are reported as the mean values. The thicknesses of the fabrics were measured using a caliper (model M500-181M, Besto Hando) at five different locations on the sample for a certain period of time (10 s) until the thickness stabilized. The standard deviation shows little variation of the measured thickness (Table 1). It should be noted that since the pressure of the caliper foot was not controlled, the comparison of the thickness between various fabrics could be difficult due to the uncertainty. The air permeabilities of the fabrics (total area = 20 cm^2^) were determined using an air permeability tester (DL-3013; Daelim Starlet Co., Ltd, Republic of Korea) at 25 Pa. Three to four measurements were taken on each of the three swatches. The porosity, pore volume, and pore size were determined using published methods^11^. Statistical analyses are provided in the Supporting Information (S2). Figure 4 shows images of the three SEM fabrics used, and Table 1 summarizes their characteristics.

### Design of a dynamic swatch test cell

**Figure 5:**
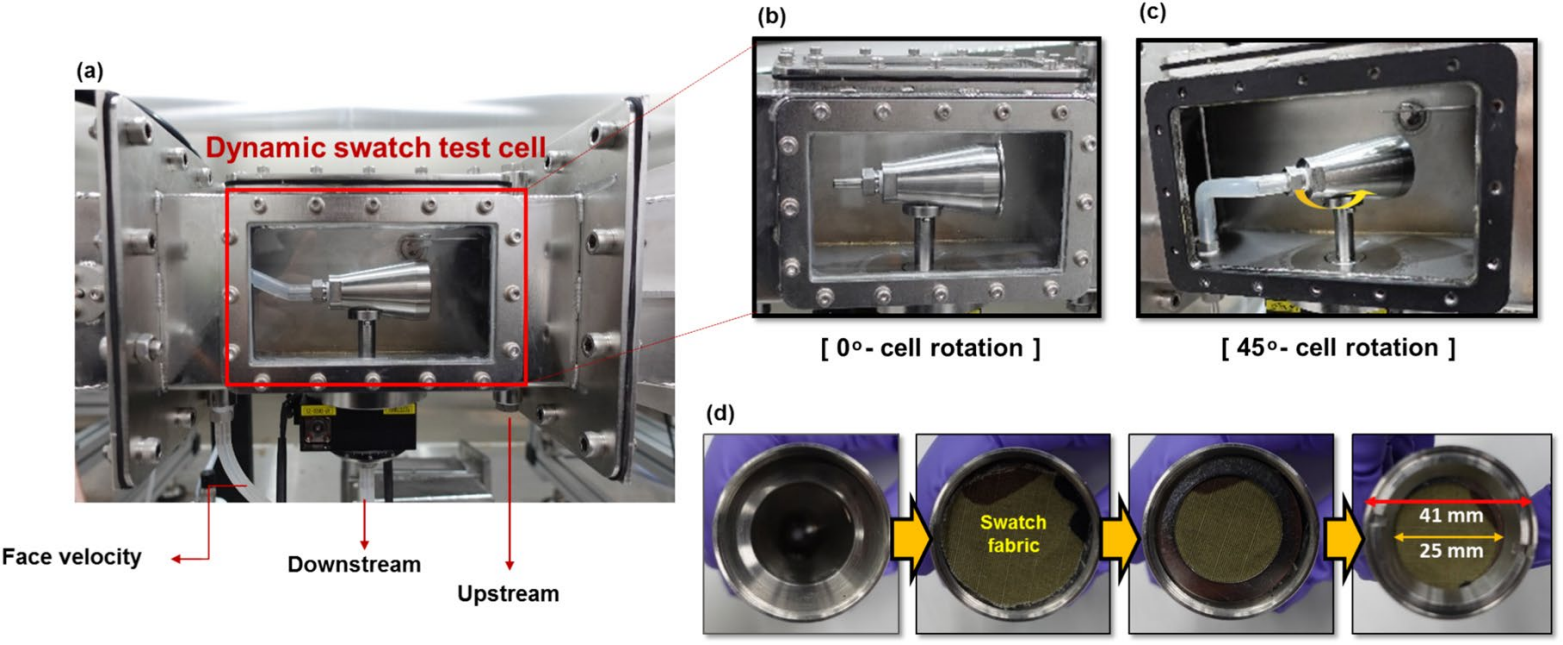
Components of the dynamic swatch test cell. (**a**) Fully assembled test cell, (**b**) 0° cell rotation, (**c**) 45° cell rotation, and (**d**) a swatch of fabric placed in the cell.

The test cell was constructed in the shape of a truncated cone with a larger anterior circle radius than the posterior radius to obtain a sufficient fabric swatch area for measuring infiltration flow (Fig. 5). Additionally, the design was intended to allow airflow and particle trajectory through and around the cell to prevent the re-entrainment of particles by turbulence, significant fluctuations in flow lines, and distortions of the particle size distribution. Air was drawn into the frusto-conical test cell from the wide front end and exited from the narrow rear end. The overall dimensions of the test cell, as shown in Fig. 5, were a maximum diameter of 41 mm at the front (with an effective sample diameter of 25 mm) and 24 mm at the back, with an overall length of 65 mm, and 20° between the back and front diameters. A flow control probe was connected to the rear. A space for an L-shaped probe was provided along the rotation axis of the dynamic cell to collect aerosol particles that penetrated the swatch.

**Figure 6:**
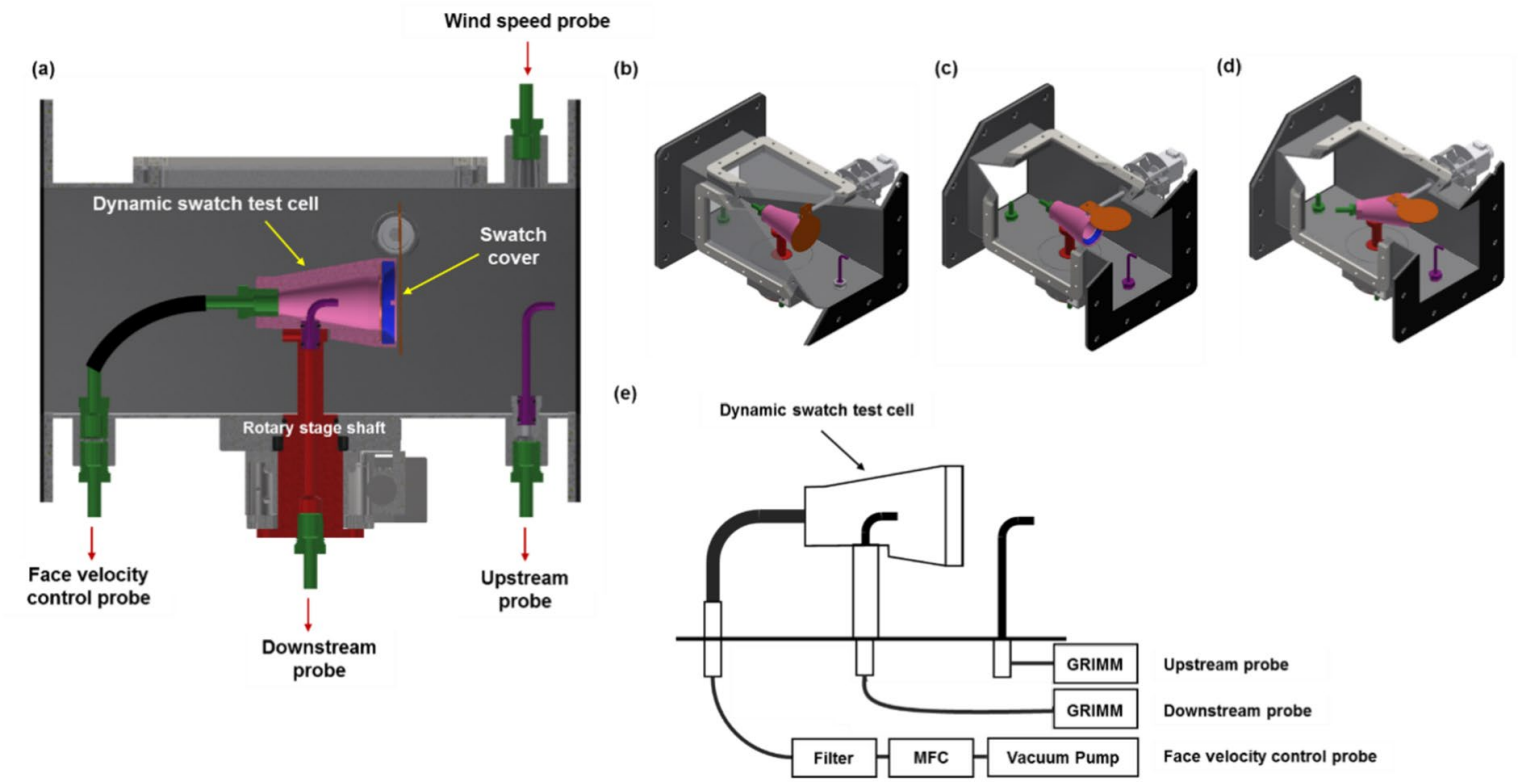
(**a**) Schematic illustration of the dynamic swatch test cell with measuring probes. (**b**) Cell with swatch cover closed, (**c**) cell with swatch cover open at 0° cell rotation, and (**d**) cell rotation at 45°.

The shaft of the rotary stage (AR-600-2S; Micro Motion Technology Co. Ltd., Republic of Korea), as shown in Fig. 6, allowed the test cell to rotate. Two M3 bolts connected the test cell to the rotary stage shaft, which was aligned with a mounting surface at the base of the test cell. The rotational motion of the dynamic cell could be aligned to rotate in one direction about the input angle and fixed at that point. Back-and-forth motion with a specific angle, such as the amplitude and input period, could also be implemented. The swatch was also able to be covered to protect the sample from aerosol particles before testing in the wind tunnel. To ensure complete coverage of the fabric sample, the circular cover was 9 mm larger than the front part of the dynamic test cell. The dynamic cover faced downward when protecting the sample. When the test began, the shaft connected to the cover and rotated 90° to move the cover upward into the wind tunnel.

Additionally, the test cell was designed to determine face velocities while the swatch was subjected to external wind speeds. A tube with a sealed end allowed for the precise and repeated delivery of a vacuum to the swatch, resulting in a reproducible face velocity, regardless of the wind speed. The flow conditions are determined by the desired face velocity, the sample cross-section, and the flow rate of the downstream sampler. Vacuum pumps were used to achieve the required flow rate. The vacuum pump mass flow rate was set using a mass flow controller (N820.3 FT.18, KNF, Germany). The vacuum pump flow rate was adjusted so that the total flow rate (outside air plus the sample probe bypass) resulted in a face velocity of 5 cm/s. The flow rate of the mass flow controllers was 0.3 L/min.

### A laboratory-sized recirculating wind tunnel

**Figure 7:**
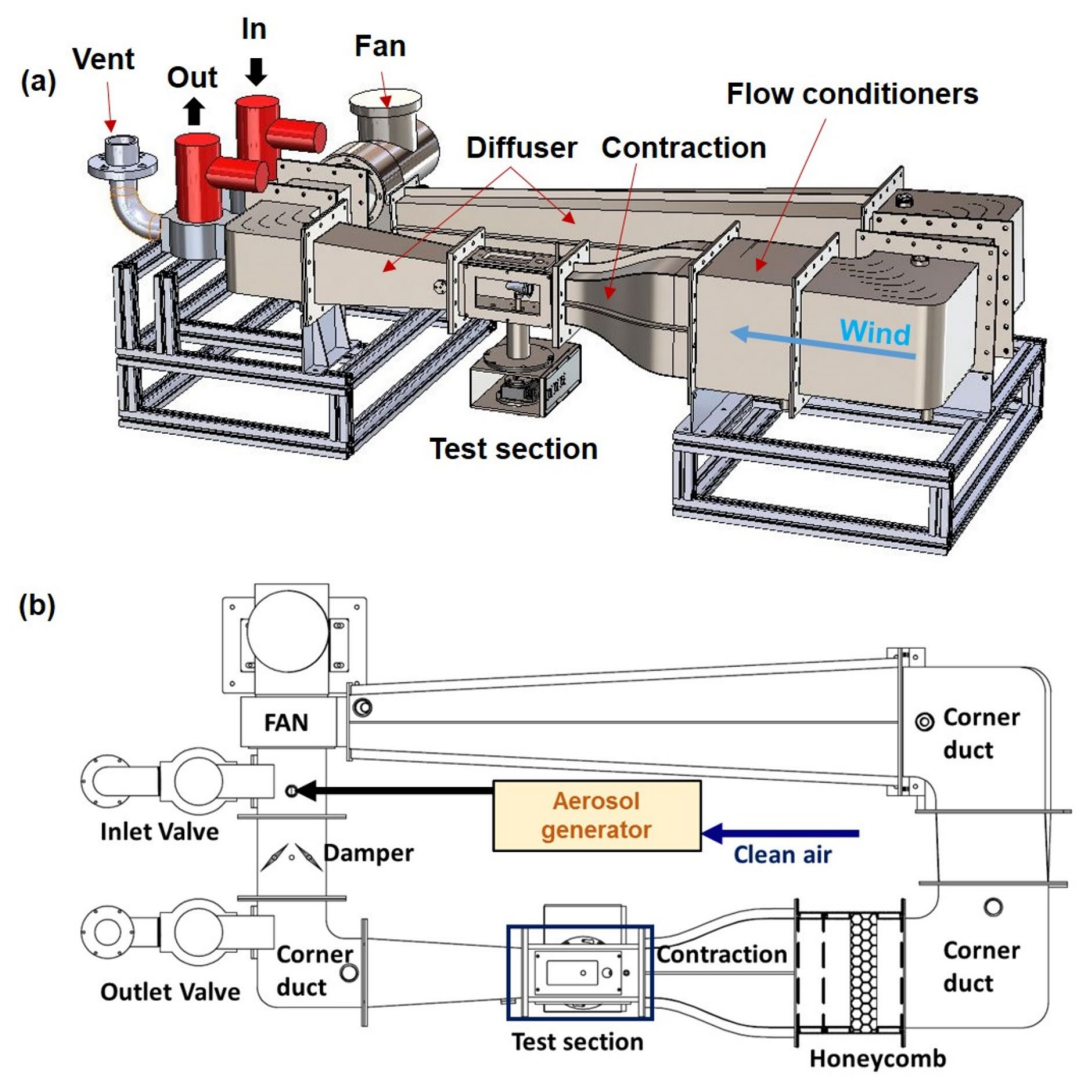
Schematic illustration of the laboratory-sized recirculating wind tunnel. (**a**) Front and (**b**) top view.

A laboratory-size recirculating wind tunnel was designed to study the penetration of particles through the chemical warfare protective clothing materials in a dynamic swatch test cell at a variety of wind speeds and relatively constant aerosol concentrations. The total dimensions of the wind tunnel are 925 mm wide, 2060 mm long, and 745 mm high, and the tunnel can be installed in a chemical fume hood in a laboratory. The test section room was 100 mm high, 100 mm wide, and 250 mm long. The tunnel was powered by a variable-speed fan with a fan drive as shown in Fig. 7 (model EXB-TB-200 TSS; INNO TECH, Republic of Korea). The tunnel had a 400 W motor driving a variable pitch fan RPM (6 CMM, 50 mmAq) at a maximum speed of 10.0 m/s. The Reynolds number (Re) was calculated to be approximately 6600 for a wind speed of 1.0 m/s. A honeycomb flow straightener was positioned approximately 350 mm upstream of the test section to reduce turbulence. Three small-mesh screens (one with 16 meshes and two with 24 meshes) were used for additional flow straightening^31^. The resulting spatial homogeneity of the velocity in the empty tunnel was approximately 0.7% (S4).

### Dynamic swatch tests

**Figure 8:**
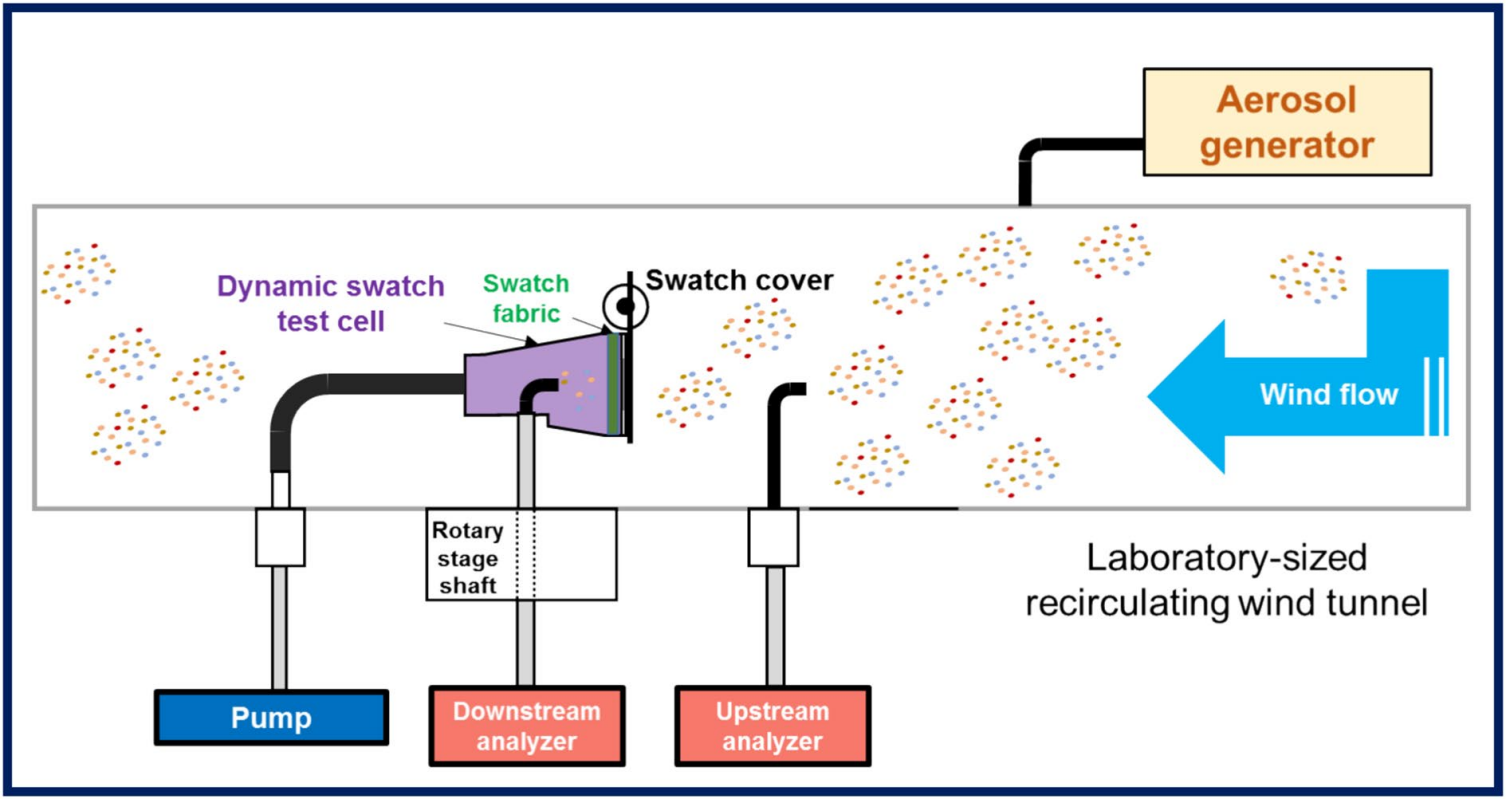
Schematic diagram of the dynamic swatch test cell setup in a laboratory-sized recirculating wind tunnel for swatch fabric penetration measurements as a function of particle size, wind speed, and cell rotation.

Figure 8 depicts the experimental setup for the dynamic swatch test in the laboratory-sized recirculating wind tunnel. A circular test swatch fabric with an exposed area^10^ of 490 mm^2^, was fixed over the opening of the particle penetration cell, which was positioned horizontally in the center of the wind tunnel test section, and the opening was perpendicular to the driving airflow. Testing was conducted in the wind tunnel under the desired test conditions after the cloth cover was opened.

A nebulizer (PLG 2000 H; Palas GmbH, Karlsruhe, Germany) generated DEHS liquid aerosols, which were passed through a dryer before being injected into the tunnel. DEHS was chosen as the chemical warfare agent simulant because this compound and *O*-ethyl *S*-2-(*N*,*N*-diisopropylamine) ethyl methyl phosphorothioate share comparable physical properties, such as viscosity and vapor pressure^32,33^. Wind tunnel speeds of 1.0, 3.0, and 5.0 m/s were used to circulate the liquid DEHS aerosols at a face velocity of 5.0 cm/s^7,10^. The wind then carried the particles to the swatch, and the upstream particle concentration was monitored using a conductive tube with a 3 mm inner diameter placed 80 mm upstream of the swatch. A vacuum pump was connected to the back of the swatch cell to generate an appropriate flow rate compatible with the desired face velocity^2^. The downstream particle concentration was sampled from inside the swatch test cell using a sample tube with a 3 mm inner diameter that was placed 18 mm downstream of the swatch. Detailed information on the particle sampling tubes and distances from the fabric swatch is provided in the supporting information (S5, S6). Two identical portable aerosol spectrometers (GRIMM 11-D; > 95% accuracy for single particle counting, DURAG GROUP, Hamburg, Germany) (S7), one (S/N: 11D21110) located 20 cm upstream of the penetration cell and the other (SN: 11D21166) connected to the downstream center of the cell, were used to measure the number and size distribution of the emitted and penetrated particles. The GRIMM 11-D detects particles from 0.253 to 35.15 μm across 31 size fractions at a temporal resolution of 6 s for 1 min. The aerosol sample flow rate was set to 1.2 L/min for both spectrometers, and simultaneous analyses showed that the exact placement of the downstream sample port within the test cell was irrelevant to the measurement of downstream concentrations.

The upstream and downstream particle size distributions were measured for particles 250–2100 nm in size (S1). The mode size of the polydisperse DEHS particles with a total concentration of 4.0–5.0 × 10^6^ particles/L was constant. This parameter varied only slightly for all tests within and between experiments. The results were reproducible and free of effects that could cause temporal variations in the data during the wind tunnel tests. The coefficient of variation (CV) of the concentration was 3%, indicating that the temporal variations had relatively stable levels (S8). Due to the size of the recirculating laboratory wind tunnel, the initial aerosol concentrations and size distributions in the tunnel approached steady-state conditions rapidly (on average, within 5 min).

Three different fabrics (A, B, and C) were examined in this study. After the wind tunnel stabilized, simultaneous DEHS particle sampling scans were performed using upstream and downstream sampling probes. Data were collected for each scan (6 s) for 1 min (total 10 scans), and a resolution of 14 channels with identical GRIMMs was obtained. The downstream sample was divided by the upstream sample to determine the degree of particle penetration^34^. The data were then plotted as penetration versus particle size.

### Statistical analysis

All data are presented as the means ± standard deviation (SDs). Groups were compared by one-way ANOVA with post hoc Tukey’s test among three samples and Student’s t-test between two samples. Statistical significance was defined as *P* < 0.05.

### Supplementary Information

Supplementary Information.

## Data Availability

The datasets used and/or analyzed during the current study available from the corresponding author upon reasonable request.
