# Supplementary material for: Dynamic swatch testing of liquid aerosols in a laboratory-sized recirculating wind tunnel
Source: Sci Rep. 2024 Jul 17;14:16539. doi: 10.1038/s41598-024-67643-0 (PMC11254901; doi:10.1038/s41598-024-67643-0)

**Supplementary Materials**

**Dynamic swatch testing of liquid aerosols in a laboratory-sized recirculating wind tunnel**

Seung Jung Yu^a^, Goonhyeok Kim^a^, Hyunsook Jung^a,b,*^, Heesoo Jung^a,*^, Jaewuk Jung^c^, and Daegyoum Kim^c^

^a^Chem-Bio Technology Center, Agency for Defense Development, Yuseong-gu, Daejeon 34063, Republic of Korea; ^b^Weapon Systems Engineering, University of Science and Technology, Gajeong-ro, Yuseung-gu, Deajeon 34113, Republic of Korea; ^c^Department of Mechanical Engineering, KAIST, Daejeon 34131, Republic of Korea

**S1. The average number of particles in the upstream and downstream in the test without fabric swatch**

The dynamic swatch test in the laboratory-sized recirculating wind tunnel was tested for 100% efficiency condition at the wind speeds of 1.0, 3.0, 3.0 with cell rotation at 45º, and 5.0 m/s. The upstream and downstream counts were recorded without swatch fabric. The average upstream and downstream DEHS aerosol counts are given as a function of particle sizes measured by GRIMMs. All experiments were repeated three times.


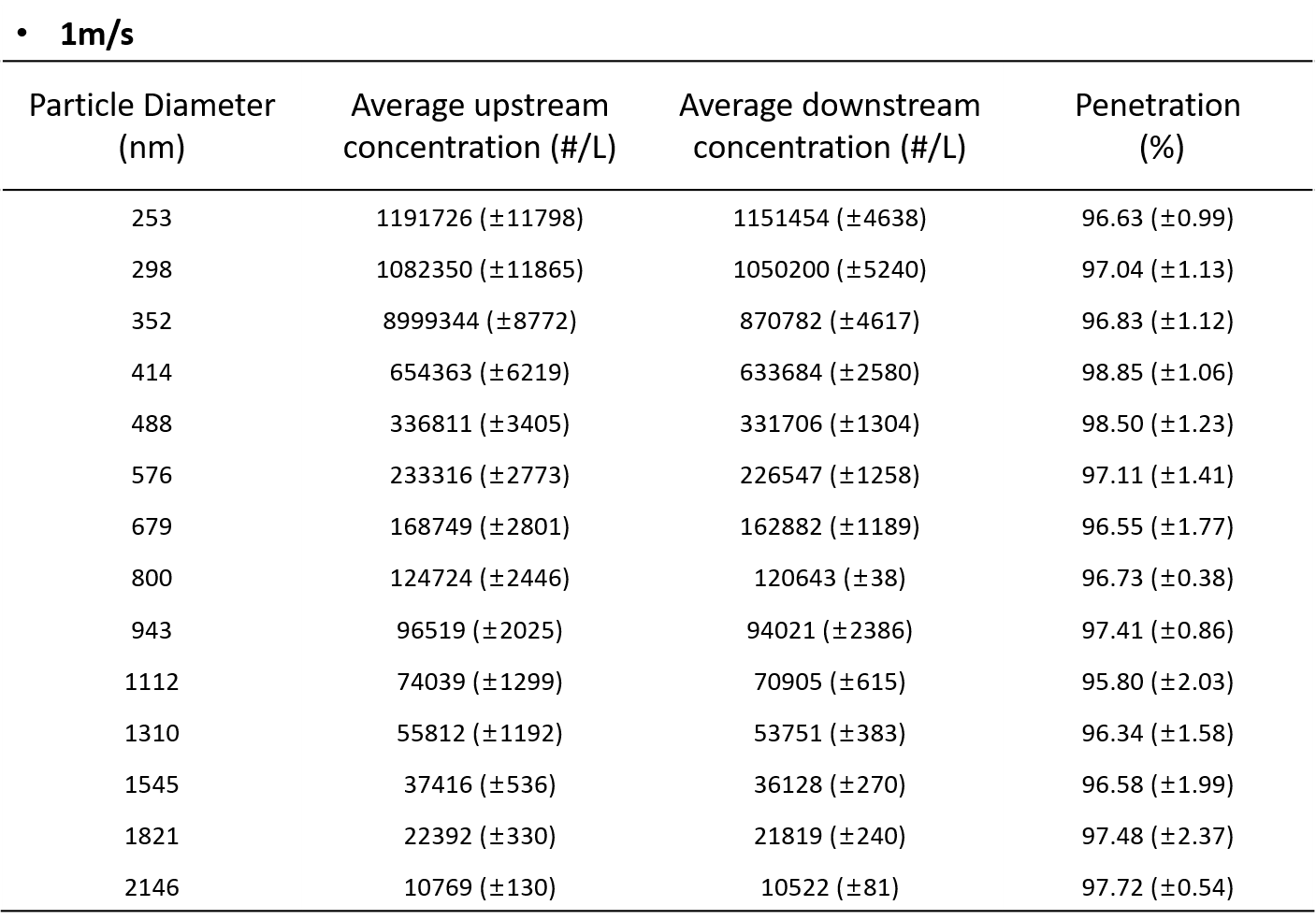


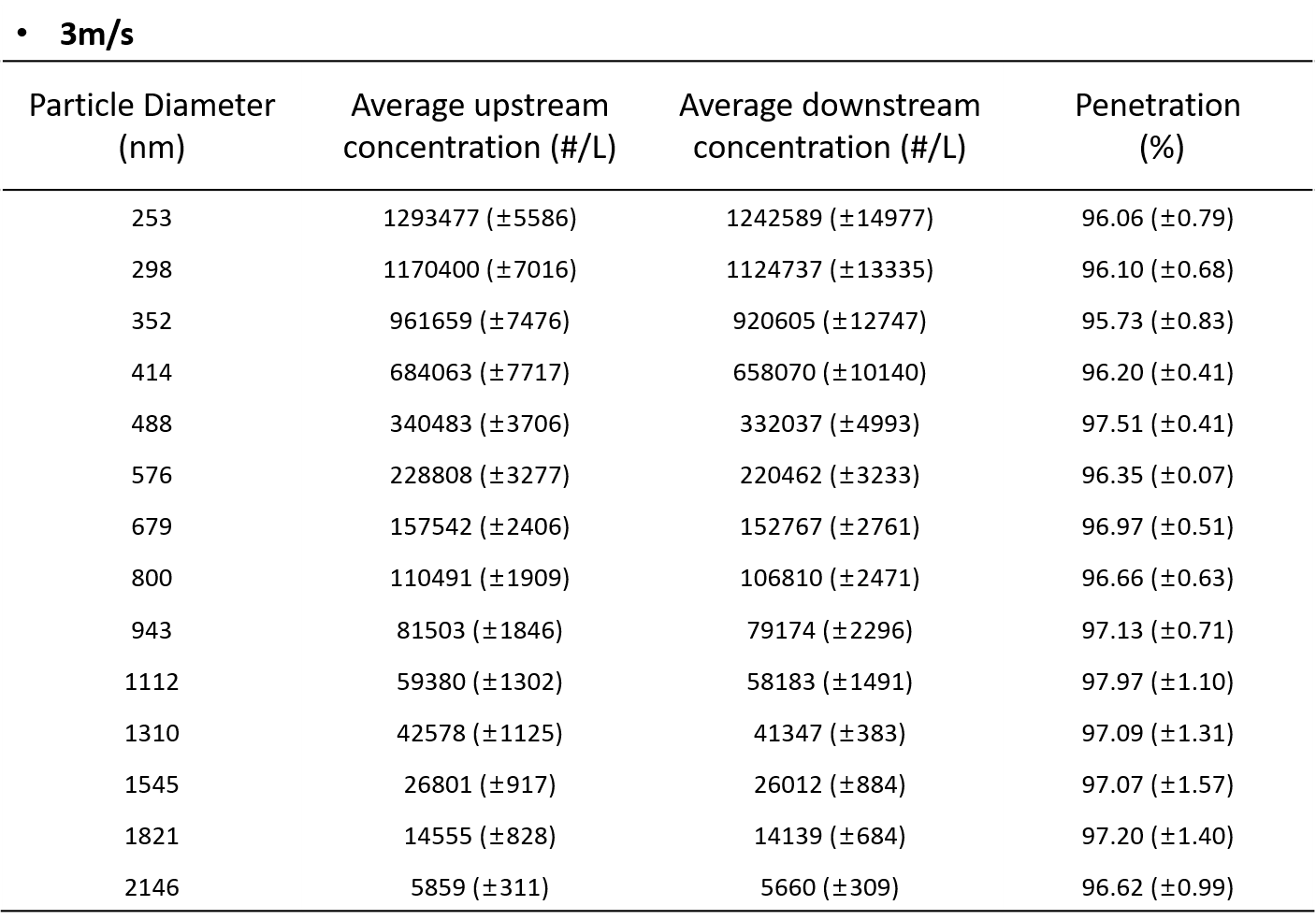


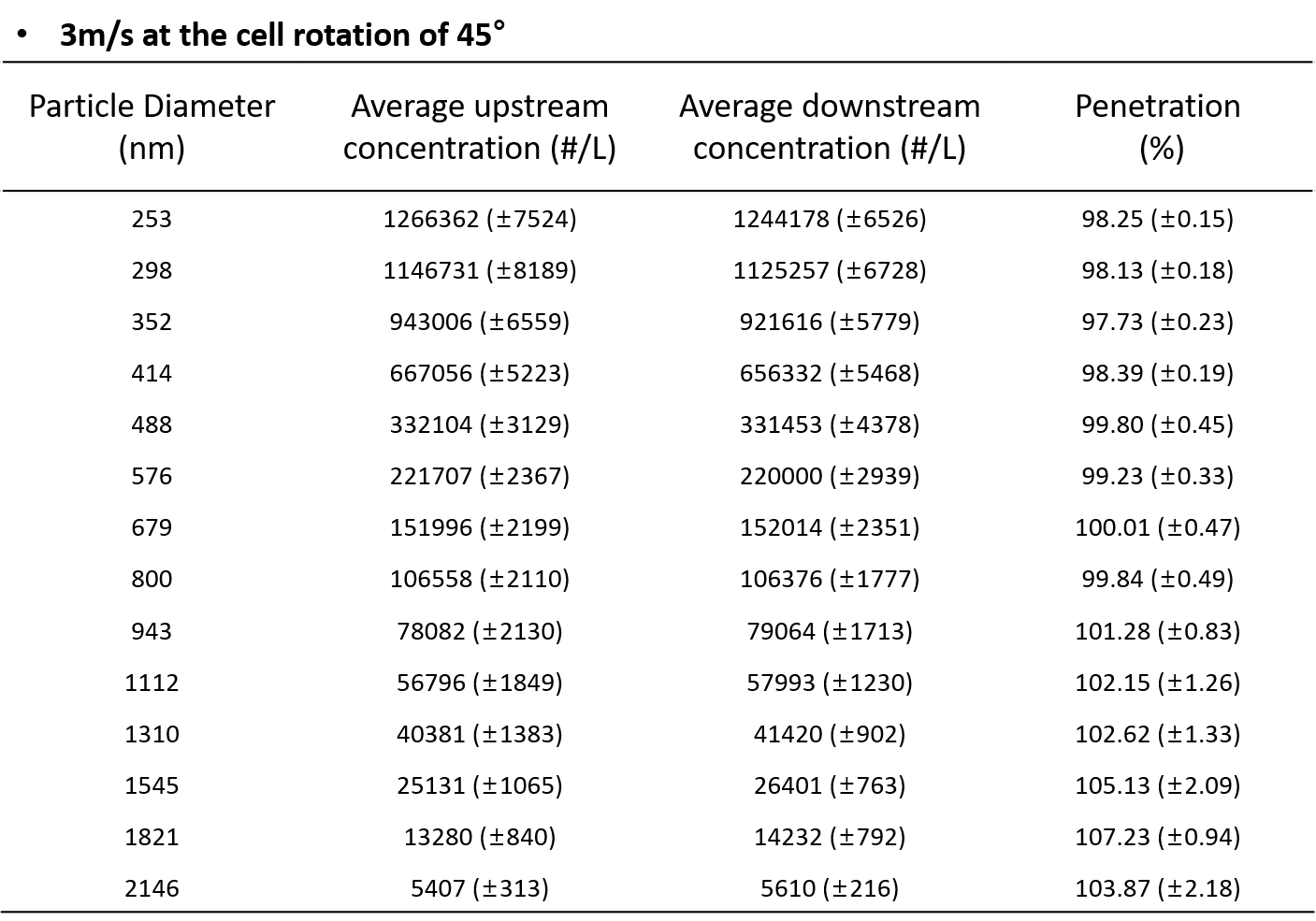


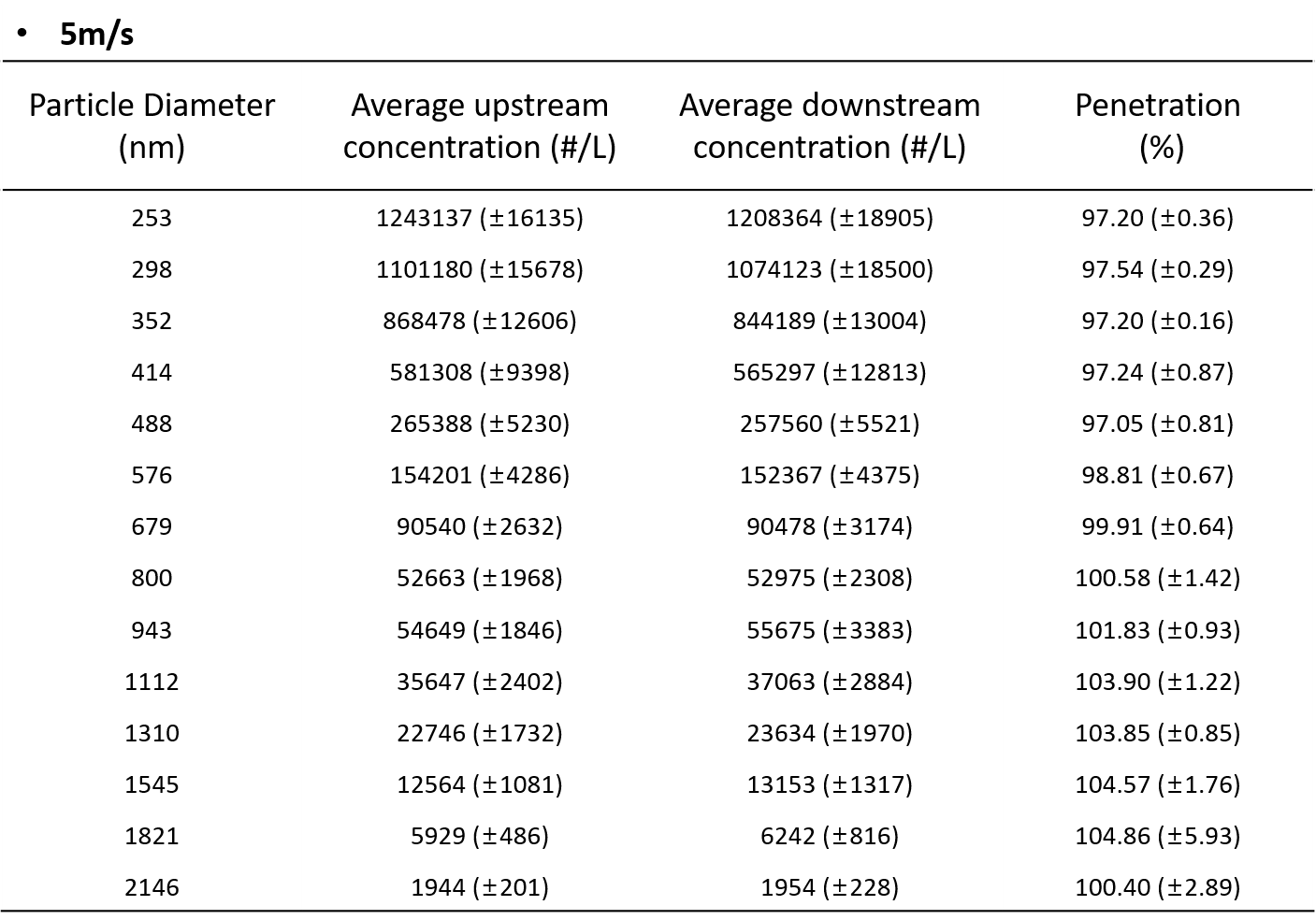


**S2. Statistical analysis for Table 1**

$\mathrm{Porosity} \left( \mathrm{dimensionless} \right) :\psi=1-\frac{\rho_{f_{a}}}{\rho_{f_{i}}}$,

$\rho_{f_{a}} is fabric density and \rho_{f_{i}} is fiber density({kg}/{m^{3}})$

$Fabric density (kg/m^{3})= \frac{W}{\mathrm{AT}}$,

W is the mass of the fabric specimen($\mathrm{kg})$, A is area of the specimen($m^{2})$ and T is the thickness of fabric ($M)$.

$Pore Volume(m^{3}/kg) : v_{s}= \frac{AT}{W}-\frac{1}{\rho_{f_{i}}}$

$Pore size\left( \mathrm{radius} \right) :r=\left[ \frac{v_{s}d}{2\pi} \right]^{\frac{1}{2}}$, d is the linear density of fiber in kg/m

Statistical analyses were conducted with one-way ANOVA with *post hoc* Tukey’s test among three fabrics (Fabric A-C) on each parameter. The statistical differences were considered to be statistically significant at a p-value less than 0.05. Statistically significant differences among three tested fabrics were observed in their thickness and air permeability.

**S3. CFD analysis on cell rotation vs. flow direction**

CFD analysis results show that particles flow perpendicular to the test for both 0º and 45º the cell rotation. This indicate that the 45º cell rotation does not reduce or increase the efficiency of the sampling probe in the downstream.

**
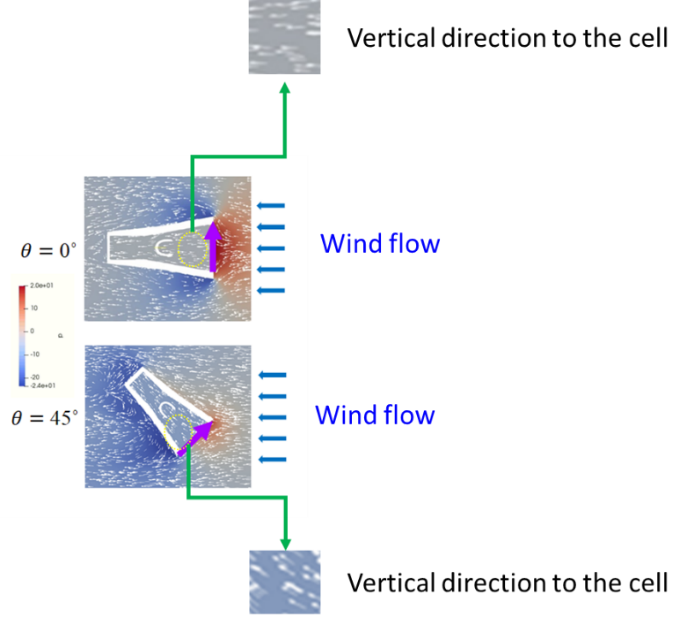
**

**S4. Spatial homogeneity of velocity**

Spatial homogeneity of velocity was analyzed according to the International standard ISO 3966 (Measurement of fluid flow in closed conduits-velocity area method using Pitot static tubes). In brief, the flow velocity was measured at each point in the test section using a hot-wire anemometer.


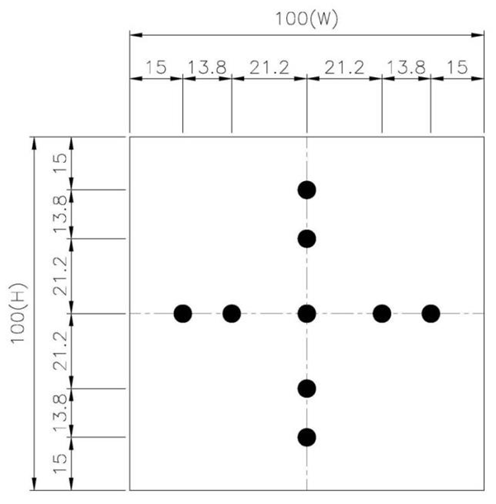


The flow velocities measured are as follows.


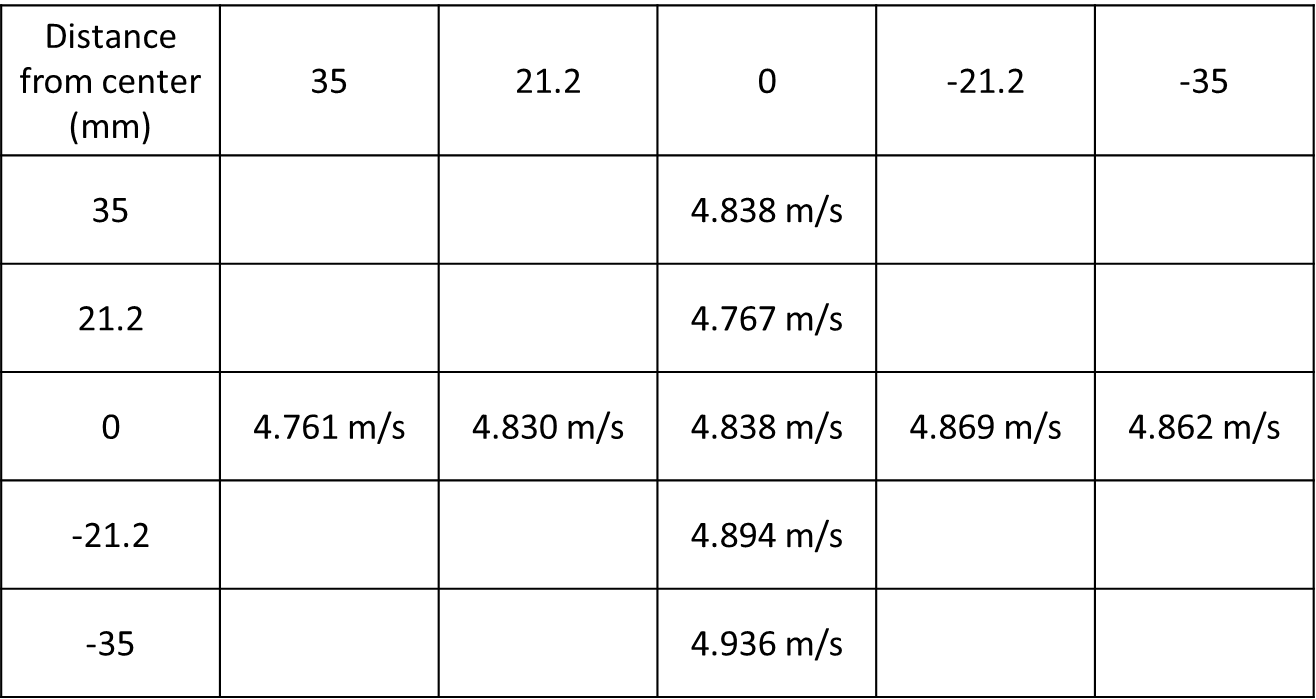


Spatial homogeneity of velocity was calculated by the following formula.

$U_{i}^{unif}=\frac{U_{i}-\overline{U}}{\overline{U}} \times100$

$U_{i}^{unif}$= Velocity uniformity at point i.

$U_{i}$= Velocity at point i

$\overline{U}$= Average velocity of all points

$\overline{U}=\frac{\sum_{i=0}^{n} U_{i}}{n}$

Spatial homogeneity of velocity is described as $max(\left| U_{i}^{unif} \right|)$. In our case, it was obtained as 0.7% in our case.

**S5. Particle sampling tube specification (length, internal diameter, and any bend)**


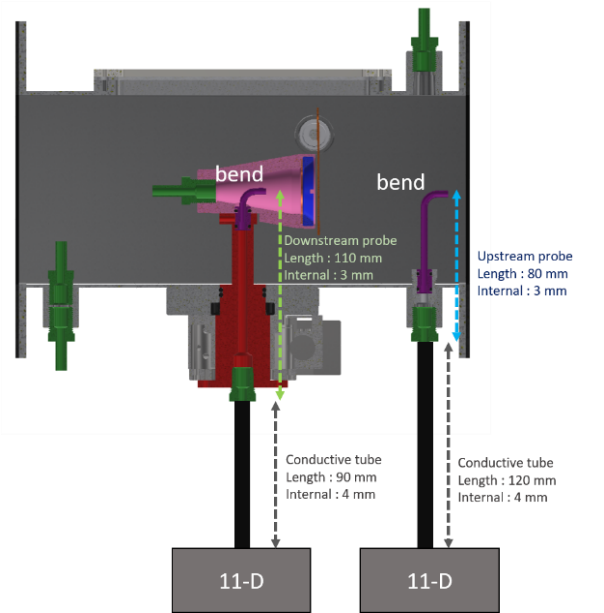


**S6. CFD analysis for upstream and downstream probes distances from the test swatch fabric**

The upstream particle concentration was monitored using a conductive tube with a 3 mm inner diameter placed 80 mm upstream of the swatch. The downstream particle concentration was sampled from inside the swatch test cell using a sample tube with a 3 mm inner diameter and placed 18 mm downstream of the swatch. These distances were selected by referring to the CFD analysis. Simulating wind speeds (1.0, 3.0, and 5.0 m/s) with and without a sampling probe at a distance of 80 mm from the swatch fabric, it was confirmed that there was almost no difference in speeds.


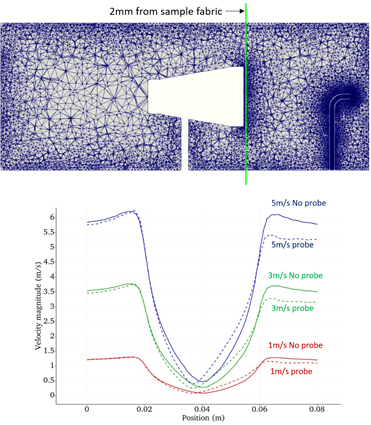


The downstream particle concentration was sampled from inside the swatch test cell using a sample tube with a 3 mm inner diameter and placed 18 mm downstream of the swatch (40 mm is now corrected as 18 mm in the text in the revised manuscript).

To decide the downstream probe distance from the fabric sample, we performed lagrangian particle tracking simulations. Under different probe position from the swatch sample (13, 18, 23 mm), aerosol diameter (1, 0.3 μm), and face velocity (5.0 cm/s), the probe distance of 18 mm has proven to measure the particle concentration in the most consistent manner. The P is collecting particle divided by total particle and the Q is collecting flow divided by total flow.


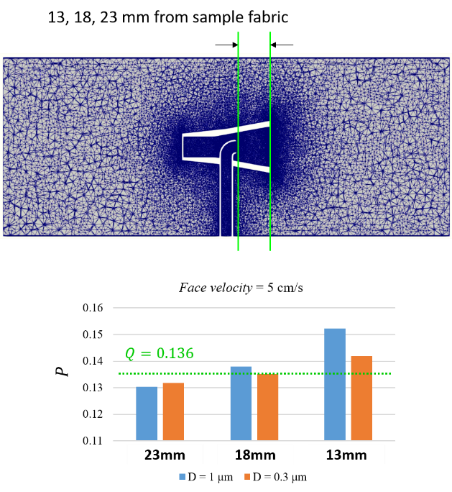


**S7. GRIMM-11D spectrometers information**


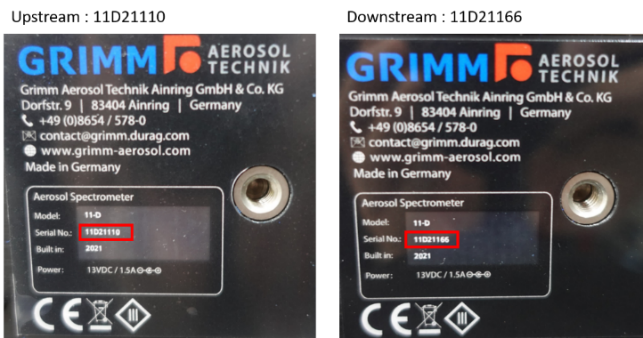


**S8. Temporal coefficient of variation (CV) of the concentration**

To quantify the amount of temporal variability, we calculated the coefficient of variation (CV = standard deviation/mean) with data measured for total concentration (6s intervals for 1 min). The CV value of 3% was obtained, indicating that the temporal variations had relatively stable levels.


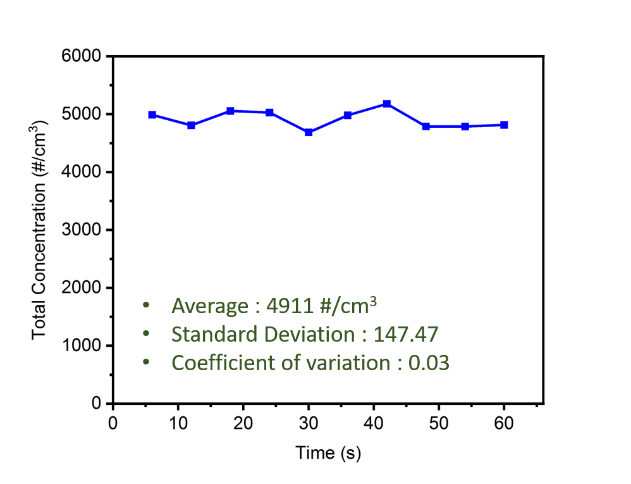


**S9. Statistical analysis for Fig. 2. Data are presented as means ± S.D. (**P*<0.05; one-way ANOVA with *post hoc* Tukey’s test)**

Statistical analyses were conducted with one-way ANOVA with *post hoc* Tukey’s test among three fabrics on each particle diameter. The statistical differences were considered to be statistically significant at a p-value less than 0.05 and marked as an asterisk on the graph (*). The statistical differences were considered to be not significant at a p-value greater than 0.05 and marked as an ‘n.s.’ on the graph.


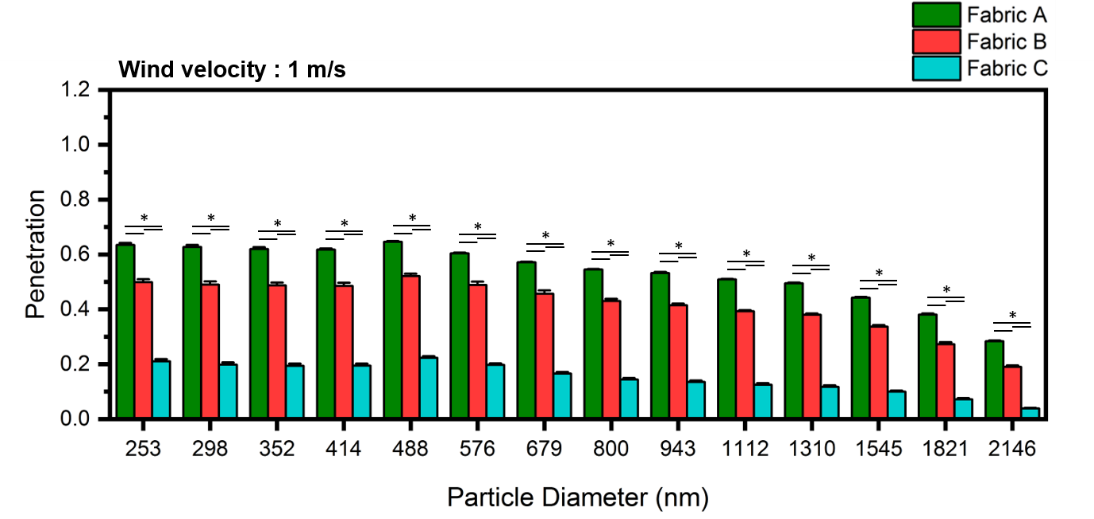


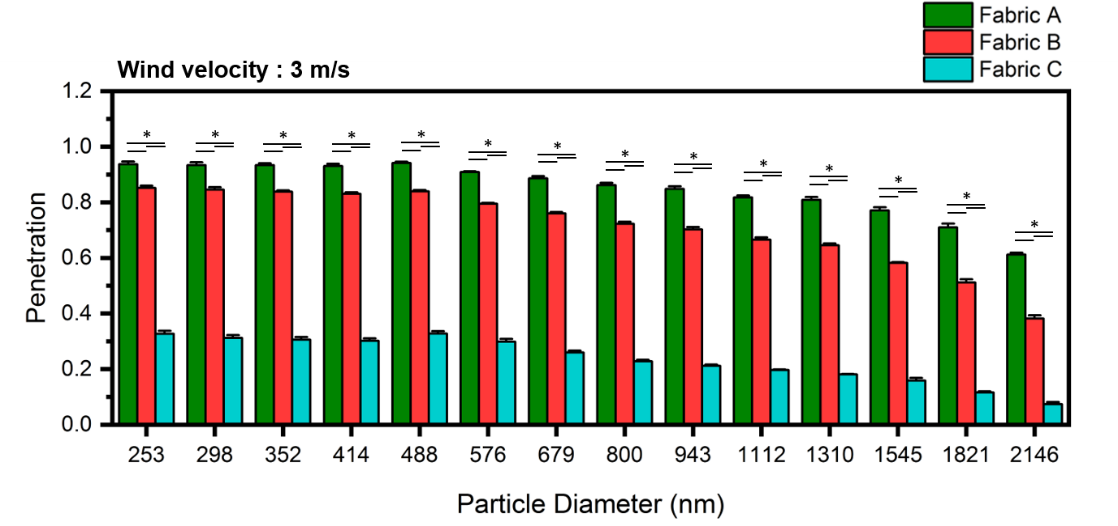


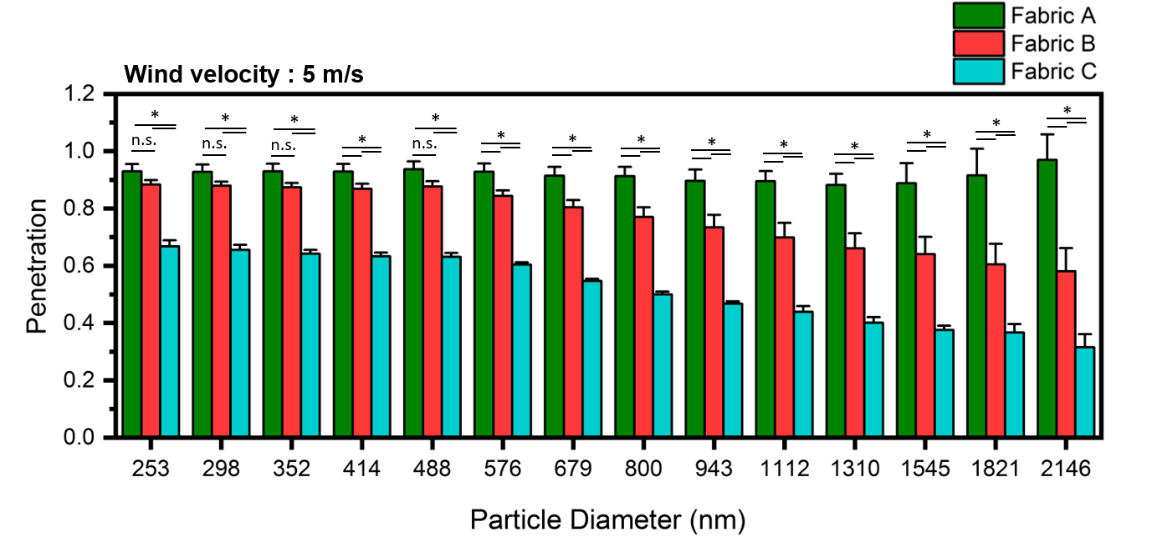


**S10. Statistical analysis for Fig. 3. Data are presented as means ± S.D. (**P*<0.05; Student *t*-test test)**

Statistical analyses were conducted with Student *t*-test between two samples on each particle diameter. The statistical differences were considered to be significant at a p-value less than 0.05 and marked as an asterisk on the graph (*). The statistical differences were considered to be not significant at a p-value greater than 0.05 and marked as an ‘n.s.’ on the graph.


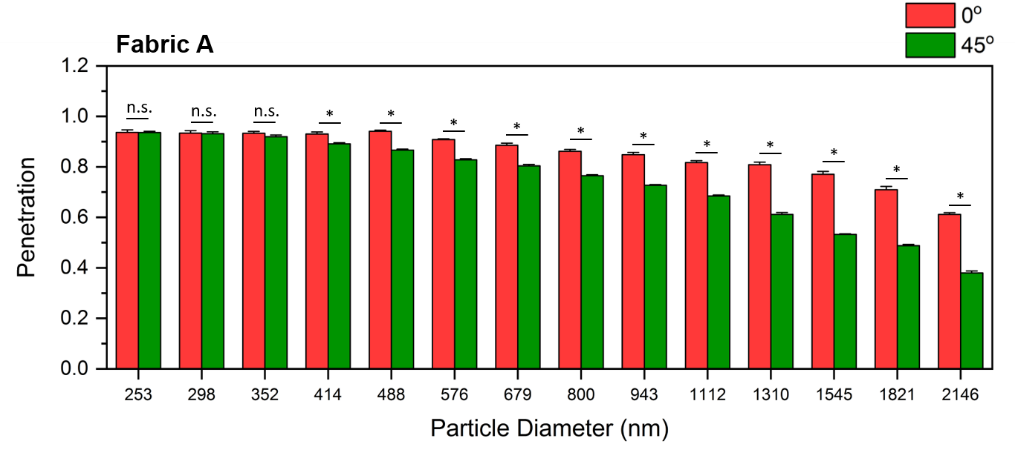


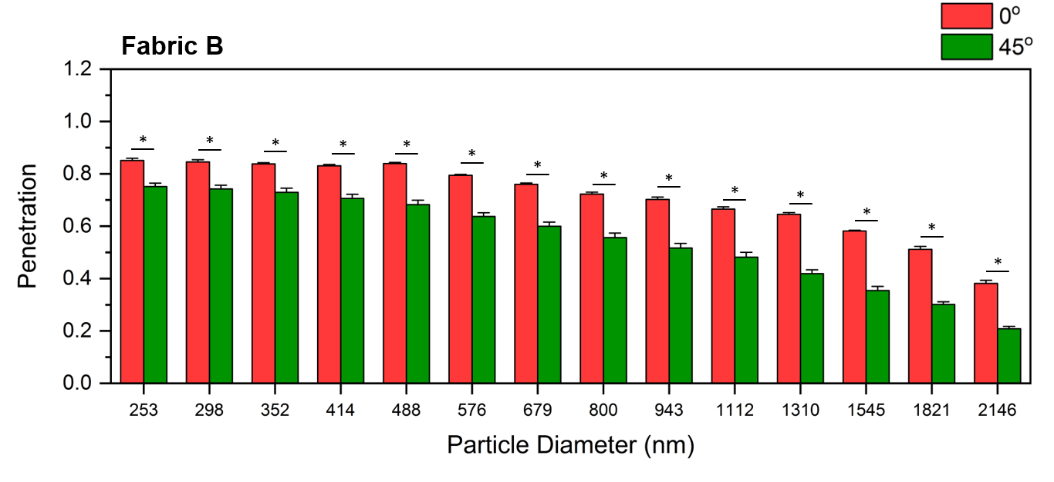


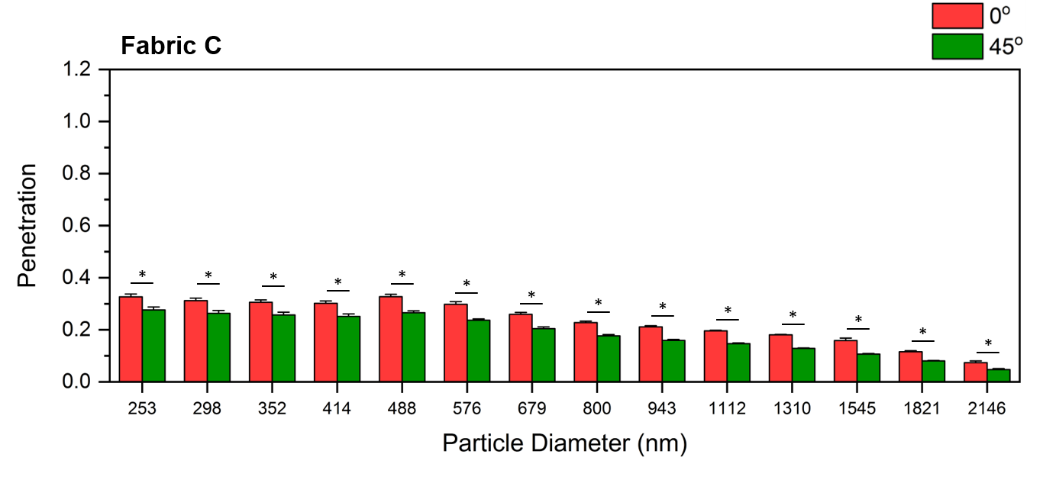


**S11. 100% penetration (without fabric) and 0% penetration (with certified N-95 masks)**


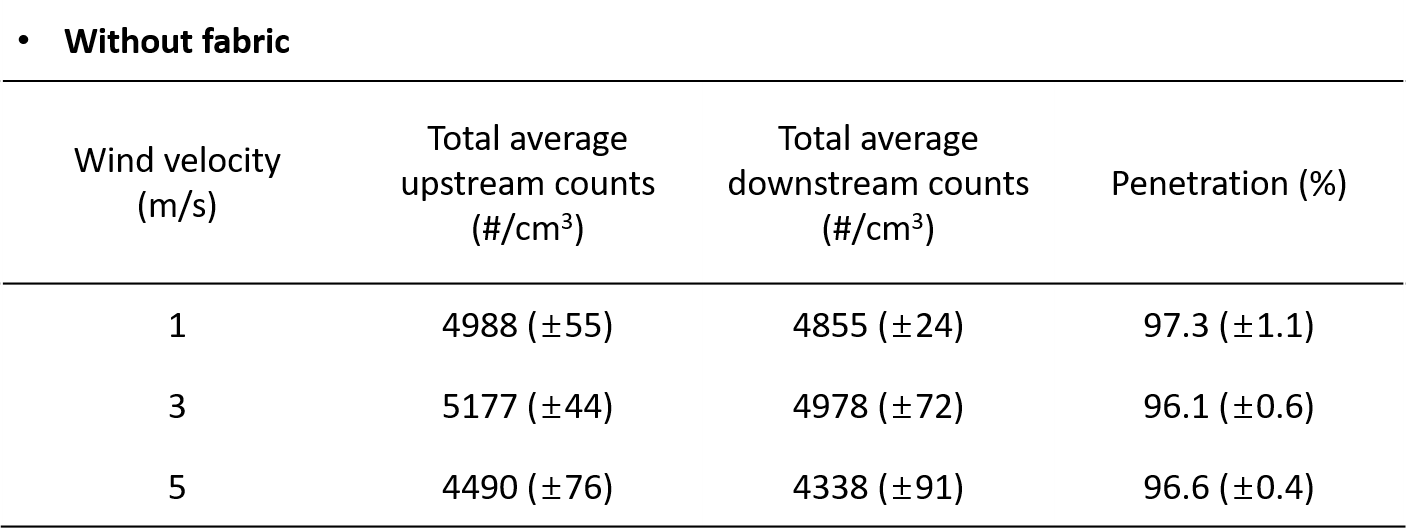


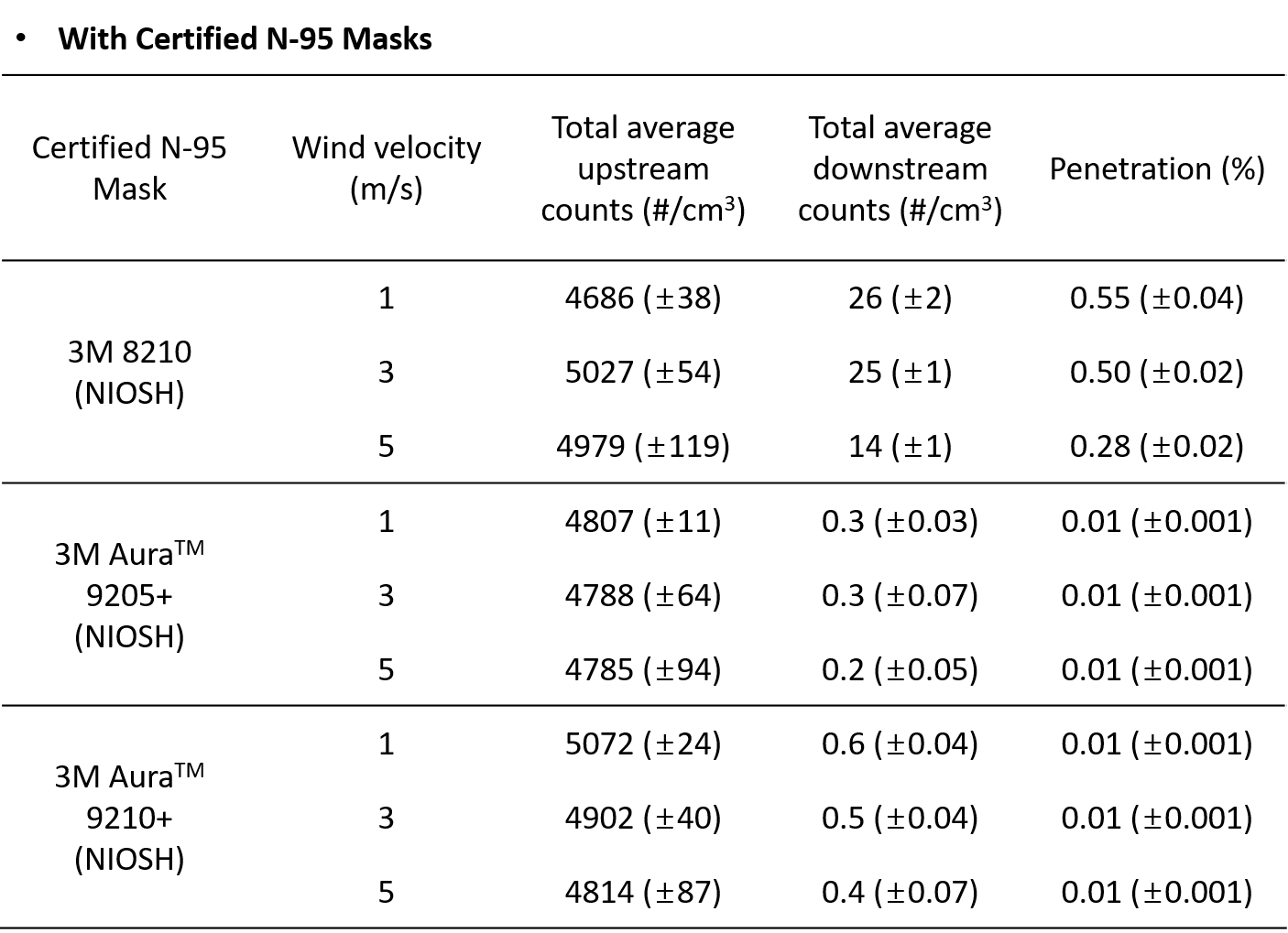


References

1. A. Kumar, D.N. Sangeetha, R. Yuvaraj, M. Menaka, V. Subramanian, and B. Venkatraman, “Quantitative performance analysis of respiratory facemasks using atmospheric and laboratory generated aerosols following with gamma sterilization”, *Aerosol and Air Quality Research*, 2020, 21(1), 200349. <https://doi.org/10.4209/aaqr.2020.06.0349>

2. A. Kumar, B. Bhattacharjee, D.N. Sangeetha, V. Subramanian, and B. Venkatraman, “Evaluation of filtration effectiveness of various types of facemasks following with different sterilization methods”, *Journal of Industrial Textiles,* 2022, 51(2_suppl), 3430S-3465S. <https://doi:10.1177/15280837211028794>

**S12. The table of all instruments used in the study and their working principles, maker, models, and limitations**


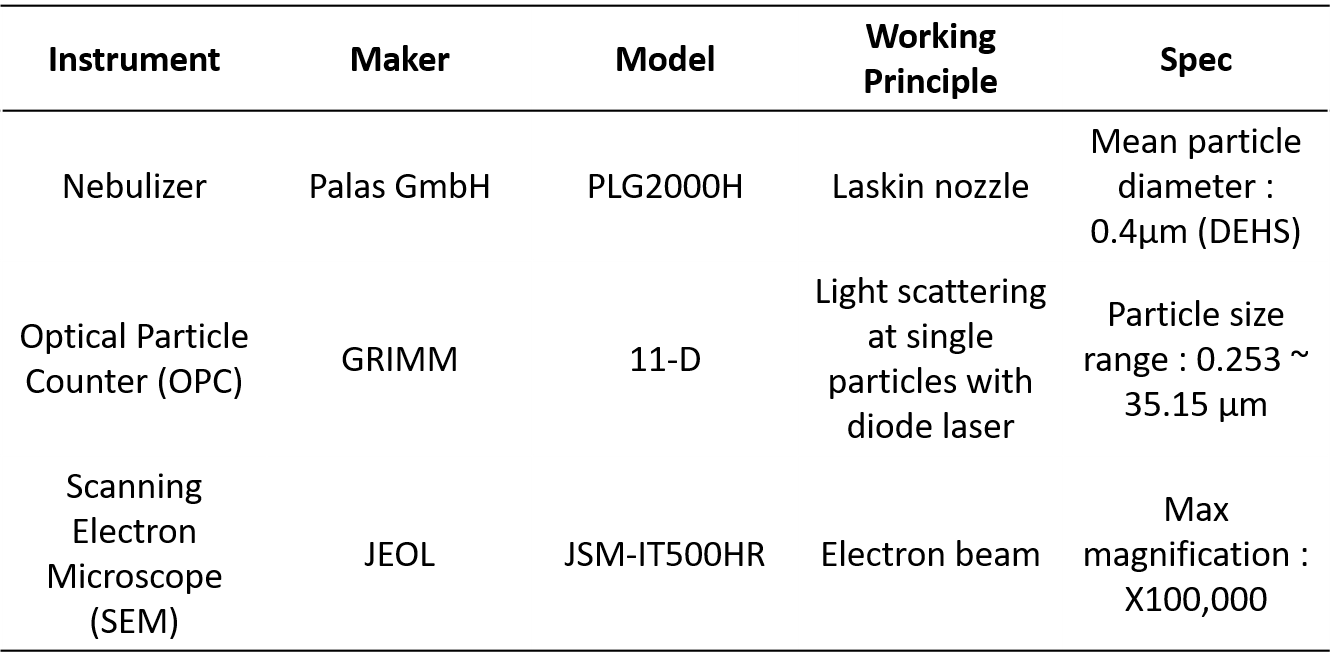

Supplement: Supplementary file 1 — Supplementary Information. [file 41598_2024_67643_MOESM1_ESM.docx]
